# A new curricular framework for an interprofessional approach to deprescribing: Why and how pharmacists should lead the way

**DOI:** 10.1177/17151635241239924

**Published:** 2024-03-15

**Authors:** Brenda G. Schuster, Sadaf Faisal, Camille L. Gagnon

**Affiliations:** Research Centre, Institut universitaire de gériatrie de Montréal

Deprescribing involves a carefully planned and supervised process of reducing or discontinuing a medication.^1^ This is typically done when a medication is causing harm or is no longer providing any benefits. Deprescribing is an important aspect of care that can reduce medication burden for patients. Furthermore, deprescribing has the potential to improve quality of life, decrease the occurrence of adverse drug events and reduce costs for both patients and the health care system.^2-4^ Pharmacists can play a crucial role in deprescribing by using their expertise in medication management and their comprehensive understanding of drug interactions and adverse effects. However, a lack of knowledge and proficiency in deprescribing has been recognized as a hurdle by numerous health care providers, including pharmacists, when it comes to putting these services into practice.^5,6^

The teaching and assessment of deprescribing skills vary across health care curricula throughout Canada, including in pharmacy.^7^ This is suboptimal, as early exposure to deprescribing education within a curriculum helps future health professionals be well equipped to navigate the evolving demands of their profession, particularly when tending to the distinct challenges posed by older, frail or complex patients. Acknowledging gaps in this area, the Canadian Medication Appropriateness and Deprescribing Network (CADeN)^8^ Health Care Provider Education Committee embarked on a collaborative journey involving professionals from diverse disciplines, including deprescribing specialists and geriatric care clinicians. Their goal was to identify the necessary competencies for acquiring deprescribing skills. Through an iterative process involving literature reviews, consultations, extensive discussions and consensus-building, this committee proposed a curriculum framework tailored to instill deprescribing competencies in entry-to-practice programs for medicine, nursing and pharmacy.^9^

The curricular framework produced contains a total of 7 proposed competencies (see BOX 1). It is structured for early, mid-level and advanced learners. Teaching and assessment strategies are also included as examples, where appropriate, for each profession. The deprescribing curriculum includes a Deprescribing Resources Toolkit that has a comprehensive list of resources about deprescribing, educational resources for teaching of deprescribing competencies, tools to identify potentially inappropriate medications, evidence-based deprescribing guidelines, publications and tools to help plan deprescribing and monitoring. Furthermore, the framework contains an implementation plan for programs to map their curriculum, identify gaps and create opportunities to integrate deprescribing education.

Implementation of a deprescribing curriculum will require the engagement of all the professions. With this new framework in hand, pharmacists’ leadership can help bring about change in schools and various care settings.

Pharmacists working in academia are encouraged over the next 2 years to map the deprescribing curriculum to determine where, when and how deprescribing competencies are included in the current programming; to determine how these competencies are taught and assessed; and to identify areas for inclusion. Subsequently, a plan to address gaps, create opportunities to implement the deprescribing competencies and determine how these will be taught and assessed would be undertaken. It is recognized that current curricula are full, but by integrating these competencies into existing educational modules that deal with prescribing competencies, instructors can adopt a structured approach to assess deprescribing knowledge. Learning activities should be active and practical, progressing from early to advanced learner skills. Integration of deprescribing teaching across the years of the academic program should be embedded in existing interprofessional education activities. Over the next 4 to 6 years, the core competencies of graduates should be evaluated to determine the effectiveness of the curricular changes, and recommendations should be made for any needed changes.

Pharmacists who serve as preceptors for pharmacy students in community, long-term care, primary care and hospital settings play a leadership role in facilitating the pharmacy student’s experiential learning of deprescribing in practice. Pharmacist preceptors must evaluate practicum students’ ability to integrate deprescribing into the medication assessment process, their use of shared decision-making with the patient, their capacity to develop care plans and their ability to collaborate with other members of the health care team. Other student activities could include reviewing a deprescribing publication, policy or resource. Additionally, student rotations would provide opportunities for special projects such as conducting education sessions on medication safety for the public, which would include the importance of medication reassessment, or creating a business plan for the development of a new clinical service related to deprescribing.

All pharmacists must demonstrate confidence in their deprescribing skills and must have knowledge of the available tools and resources to guide students. The competencies outlined in the proposed curriculum can serve as a self-assessment tool for pharmacists to identify gaps in their knowledge or skills related to deprescribing. Assessing opportunities for deprescribing, engaging in shared decision-making, implementing the deprescribing plan and following up are skills we must demonstrate and evaluate with our learners. Pharmacists who want to advance their skills can access the Deprescribing Resources Toolkit (https://link.springer.com/article/10.1007/s40670-022-01704-9/tables/3).

**Box 1 Seven key competencies for guiding the deprescribing process**

The Deprescribing Competency Curriculum Framework outlines 7 key competencies and associated skills essential for effectively guiding the deprescribing process across entry-to-practice, mid-level and advanced learners within the disciplines of medicine, nursing and pharmacy. The identified competencies are the following:

1. Conducting a comprehensive patient medication history.
2. Interpreting relevant information in the context of desired therapeutic outcomes and goals of care.
3. Identifying medications that are no longer necessary, may have more harm than benefit or are otherwise potentially inappropriate.
4. Assessing the deprescribing potential of each medication by weighing benefits and harms.
5. Deciding whether deprescribing a medication is appropriate, using shared decision-making.
6. Designing, documenting and sharing a deprescribing and monitoring plan.
7. Monitoring patient progress and providing support.

A knowledge mobilization plan has been developed to strategically connect the various health care professionals, academic institutions and health care organizations with the deprescribing curriculum framework, resources and tools. We encourage shared learnings of curricular innovations, professional development opportunities, prescribing and deprescribing competency frameworks with the CADeN team (info@caden-recad.ca) to facilitate dissemination, networking and collaboration across Canada.

The new curricular framework provides a comprehensive approach for the professions to come together to educate our future health care professionals. The deprescribing process is an integral part of prescribing and an important part of the medication reassessment process. Building deprescribing skills and knowledge is essential to ensure that current and future pharmacists are able to deprescribe safely, with the end goal to minimize the personal and societal costs of medication-related harm.

## References

[1] Reeve E Shakib S Hendrix I Roberts MS Wiese MD. Review of deprescribing processes and development of an evidence-based, patient-centred deprescribing process. Br J Clin Pharmacol 2014;78(4):738-47. doi:10.1111/bcp.12386 PMC4239968 24661192

[2] Reeve E Thompson W Farrell B. Deprescribing: a narrative review of the evidence and practical recommendations for recognizing opportunities and taking action. Eur J Intern Med 2017;38:3-11. 28063660 10.1016/j.ejim.2016.12.021

[3] Moriarty F Cahir C Bennett K Fahey T. Economic impact of potentially inappropriate prescribing and related adverse events in older people: a cost-utility analysis using Markov models. BMJ Open 2019;9:e021832. doi:10.1136/bmjopen-2018-021832 PMC6359741 30705233

[4] Sanyal C Turner JP Martin P Tannenbaum C. Cost-effectiveness of pharmacist-led deprescribing of NSAIDs in community-dwelling older adults. J Am Geriatr Soc 2020;68:1090-7. doi:10.1111/jgs.16388 32105355

[5] Farrell B Richardson L Raman-Wilms L de Launay D Alsabbagh MW Conklin J. Self-efficacy for deprescribing: a survey for health care professionals using evidence-based deprescribing guidelines. Res Social Adm Pharm 2018;14:18-25. doi:10.1016/j.sapharm.2017.01.003 28214150

[6] Anderson K Stowasser D Freeman C Scott I. Prescriber barriers and enablers to minimising potentially inappropriate medications in adults: a systematic review and thematic synthesis. BMJ Open 2014;4:e006544. doi:10.1136/bmjopen-2014-006544 PMC4265124 25488097

[7] Raman-Wilms L Farrell B Sadowski C Austin Z. Deprescribing: an educational imperative. Res Social Adm Pharm 2019;15:790-5. doi:10.1016/j.sapharm.2018.08.011 30241871

[8] The Network. Do I still need this medication? Is deprescribing for you? 2023.Available: https://www.deprescribingnetwork.ca/canadian-deprescribing-network (accessed Oct. 15, 2023).

[9] Farrell B Raman-Wilms L Sadowski CA , et al. A proposed curricular framework for an interprofessional approach to deprescribing. Med Sci Educ 2023;33(2):551-67. doi:10.1007/s40670-022-01704-9 PMC10226933 37261023

